# Relationships between anaemia and parasitic infections in Kenyan schoolchildren: A Bayesian hierarchical modelling approach

**DOI:** 10.1016/j.ijpara.2008.05.013

**Published:** 2008-12

**Authors:** Artemis Koukounari, Benson B.A. Estambale, J. Kiambo Njagi, Bonnie Cundill, Anthony Ajanga, Christopher Crudder, Julius Otido, Matthew C.H. Jukes, Siân E. Clarke, Simon Brooker

**Affiliations:** a Schistosomiasis Control Initiative, Department of Infectious Disease Epidemiology, Faculty of Medicine, Imperial College, Norfolk Place, London W2 1PG, UK; b Institute of Tropical and Infectious Diseases, University of Nairobi, Nairobi, Kenya; c Division of Malaria Control, Ministry of Health, Nairobi, Kenya; d Department of Infectious and Tropical Diseases, London School of Hygiene and Tropical Medicine, Keppel Street, London WC1E 7HT, UK; e Harvard Graduate School of Education, Harvard University, Cambridge, MA, USA; f Malaria Public Health and Epidemiology Group, KEMRI/Wellcome Trust Collaborative Programme, Nairobi, Kenya

**Keywords:** Anaemia, Haemoglobin concentration, Malaria, Helminth infections, Bayesian hierarchical models, School children, Kenya

## Abstract

Anaemia is multi-factorial in origin and disentangling its aetiology remains problematic, with surprisingly few studies investigating the relative contribution of different parasitic infections to anaemia amongst schoolchildren. We report cross-sectional data on haemoglobin, malaria parasitaemia, helminth infection and undernutrition among 1523 schoolchildren enrolled in classes 5 and 6 (aged 10–21 years) in 30 primary schools in western Kenya. Bayesian hierarchical modelling was used to investigate putative relationships. Children infected with *Plasmodium falciparum* or with a heavy *Schistosoma mansoni* infection, stunted children and girls were found to have lower haemoglobin concentrations. Children heavily infected with *S. mansoni* were also more likely to be anaemic compared with uninfected children. This study further highlights the importance of malaria and intestinal schistosomiasis as contributors to reduced haemoglobin levels among schoolchildren and helps guide the implementation of integrated school health programmes in areas of differing parasite transmission.

## 1 Introduction

Anaemia remains one of the most intractable public health problems in Africa, contributing to a quarter of Africa’s nutrition-related Disability Adjusted Life Years (DALYs) lost (World Health Organization, 2002). Several studies have highlighted the contribution of parasitic diseases to childhood anaemia. Recent meta-analyses of malaria intervention trials among African children, for example, provide compelling evidence that both symptomatic and asymptomatic malaria contributes to anaemia (Geerligs et al., 2003; Korenromp et al., 2004). The effect of hookworm infection is also well documented, with risk of anaemia correlated with intensity of infection (Stephenson, 1993; Olsen et al., 1998; Brooker et al., 1999); in contrast, the contributory role of schistosomiasis remains unclear (Desai et al., 2005b; Friedman et al., 2005a). However, there are surprisingly few published studies describing the relative contribution of these different parasitic infections in populations of school-aged children (Olsen et al., 1998; Tatala et al., 1998; Friis et al., 2003; Leenstra et al., 2003; Leenstra et al., 2004; Desai et al., 2005a; Friedman et al., 2005b). Investigation of this issue is particularly relevant for the design of integrated control strategies aimed at reducing anaemia, including anthelmintic treatment programmes, micronutrient supplementation and malaria control measures, through school health programmes. In particular, the same suite of school-based interventions will not be relevant everywhere, and the selection of intervention options will need to be guided by an informed understanding of the epidemiology of parasite-related anaemia (Crawley, 2004), as well as of the geography of infection (Brooker et al., 2007).

The aim of the present study was to examine the relationships of haemoglobin (Hb) concentration and anaemia with common parasitic infections, including malaria, hookworm, *Ascaris lumbricoides*, *Trichuris trichiura* and *Schistosoma mansoni* in school children in western Kenya through Bayesian hierarchical modelling. Adjustment for nutritional and socioeconomic status (SES) has also been taken into account here as they might influence anaemia risk (Ong’echa et al., 2006). By employing a Bayesian approach for the statistical modelling of the Hb counts and of anaemia prevalence, our model specification via Markov chain Monte Carlo (MCMC) algorithms offers flexibility in fitting complex models and enables estimates for the whole distribution of the unknown parameters, including point and interval estimates, to be derived. This approach is in contrast to the frequentist approach which often only gives estimates and crude standard errors based on asymptotic results.

## 2 Materials and methods

### 2.1 Study area

The study was conducted between February and March 2005 in 30 primary schools in Bondo district in western Kenya. Malaria transmission is intense and perennial (Beier et al., 1994), with two seasonal peaks, March–May and November–December, following the long and short rainy seasons, respectively. Previous studies in western Kenya have reported a high prevalence of hookworm and *T. trichiura* infections and a medium prevalence of *S. mansoni* and *A. lumbricoides* infections (Brooker et al., 2000; Thiong’o et al., 2001).

### 2.2 Study design

This study used cross-sectional, baseline data from a stratified, cluster-randomised placebo-controlled trial of the impact of antimalarial intermittent preventive treatment (IPT) among schoolchildren. The trial design and protocol are described elsewhere (Clarke et al., 2008). Briefly, sample size was estimated on the basis of the expected impact of IPT on anaemia, using the methods for cluster-randomised trial design proposed by Hayes and Bennett (1999). The 30 study schools were randomly selected from primary schools in Usigu and Maranda Divisions with ⩾150 pupils with >15 pupils per class and located more than 5 km from the shores of Lake Victoria, so as to minimise the effect of *S. mansoni* which is generally only prevalent along the shoreline (Brooker et al., 2001; Handzel et al., 2003). No stratification by intestinal nematode was undertaken because of their relatively homogeneous distribution (Handzel et al., 2003) but schools were stratified according to past school examination performance. We present data from the baseline survey on a sub-sample of children enrolled in classes 5 and 6 (age range 10–21 years) for whom complete data on anaemia, helminth infection, malaria parasitaemia, nutritional status and SES were available.

### 2.3 Procedures

Finger-prick blood samples were obtained from all children to assess Hb levels and malaria parasitaemia. Haemoglobin was measured in the field using a portable photometer (Haemocue, Angelholm, Sweden). Malaria parasite prevalence and parasite densities were estimated in Giemsa-stained thick blood films, assuming an average white blood cell count of 8,000 per μl, with species identification carried out on Giemsa-stained thin films. A slide was declared negative after examination of 100 high-powered fields. Stool samples provided by each child were examined microscopically using the semi-quantitative Kato-Katz technique and intensity of infection was expressed as eggs/gram of faeces. Height was measured to the nearest 0.1 cm using a Leicester portable fixed base stadiometer (Chasmors, UK) and weight was measured to the nearest 0.1 kg using an electronic balance. A simple questionnaire was administered to pupils to obtain data on key socio-economic variables including: structure of the house, type of overall light, ownership of bicycle, use of bednet as well as education of the child’s guardian.

Ethical clearance for the study was obtained from the ethics committee of the Kenyatta National Hospital, Kenya and from the London School of Hygiene and Tropical Medicine, UK. Permissions were obtained from the Ministry of Education, and the district education and health authorities, and headteachers. Prior to the start of the study, a series of meetings were held in participating schools to explain the nature and purpose of the trial and to obtain individual informed parental consent from the parents or legal guardians of children enrolled in study schools.

### 2.4 Statistical analysis

An index of SES was constructed from asset and education variables using principal component analysis (Filmer and Pritchett, 2001). Data were available for 1453 (92%) of the 1577 children in 30 schools. Analysis was done using the PROC PRINCOMP command in SAS version 9.1 (SAS Institute Inc., Cary, NC). For the index of SES, the first principal component explained 28% of the variance in the asset and education variables with the greatest weight given to the presence of a permanent house structure (0.43), and the lowest weight to the presence of a traditional house structure (−0.39), respectively. Weights for each variable were derived from the first principal component and applied to each child to derive a SES index. We then assigned these children to a group on the basis of their value on the index. Following the approach of Filmer and Pritchett, we classified children into a 0–39 percentile, 40–79 percentile and upper 20 percentile, which we refer to as ‘most poor’, ‘poor’ and ‘least poor’, respectively. Anthropometric indices were calculated on the basis of the 2000 Centres for Disease Control and Prevention (CDC) Growth Charts, and analysed as binary variables. Children were classified as stunted if z-scores of height-for-age were less than 2 S.D. below the CDC median. Body mass index, which is weight (kg)/height (cm)^2^, was also calculated and a cut-off of −2 Body Mass Index *Z*-scores (BMIZ) were calculated to classify underweight children.

Data management and bivariate relationships between mean Hb concentration and key predictors examined were obtained using SAS V 9.1 (SAS Institute Inc., Cary, NC, USA).

#### 2.4.1 Bayesian modelling

Bayesian hierarchical modelling was used to assess associations between anaemia, Hb and parasitic infection, including children and schools as random effects drawn from some common prior distribution with unknown parameters. Children within each school as well as the results from different schools were treated as ‘exchangeable’ in the sense that their joint probability densities should be invariant to permutations of the indexes (Gelman et al., 2004).

In Bayesian analysis, the proposed model of the observed data is combined with the prior distribution of all the unknown model parameters to give the posterior distributions for all unknowns. MCMC methods (Gilks et al., 1996) are used to sample from the posterior distributions of the unknown parameters. Analysis was conducted using WinBUGS which employs the Gibbs sampler to form the posterior distribution for each unknown parameter by drawing samples from their full conditional distributions (Spiegelhalter et al., 2003) to fit models. An advantage of the Bayesian hierarchical approach is that prior information can be incorporated in the model in probabilistic form. However, in the absence of any prior knowledge about the model parameters, the choice of non-informative improper priors is dictated by pragmatic conditions (Diggle et al., 2002). Model convergence was evaluated on the basis of inspection of sample traces, which all showed a reasonable degree of convergence to a stationary distribution.

We fitted Bayesian normal hierarchical models on Hb because there is a two-level data structure applied to cross-sectional data. Individual subjects were classified at the lower level for older children with data on helminth infection and age range 10–21 years old was classified by cluster at the higher level. We assume that individual *i* (=1,…,*n*) – where *n* = 1523 can belong in any of *j* (=1,…,*n*2) – where *n*2 = 30 schools. Hb at a child level may be affected by those childrens’ characteristics (age, sex, intensities of helminth infections, nutritional and socio-economic status) but may also vary according to which school these children are enrolled in. Specifically for Hb, we used a random intercepts normal model which can be written as:

$$\begin{array}{cc} & {\mathrm{Hb}}_{\mathrm{ij}}∼\text{normal}(\mu_{\mathrm{ij}}\text{,}\tau )\text{,}\\ & \mathrm{with}\;\mu_{\mathrm{ij}}=X_{\mathrm{ij}}\beta +u_{j}\;\mathrm{and}\;\tau ∼\text{gamma}(0.001\text{,}0.001)\text{,}\\ & u_{j}∼\text{normal}(0\text{,}\tau_{u_{j}})\;\mathrm{and}\;\tau_{u_{j}}∼\text{gamma}(0.001\text{,}0.001)\text{.} \end{array}$$

Where *μ*_*ij*_ is given by the sum of the product of *X*_*ij*_ with *β* – this constitutes the fixed part of the model, and *u*_*j*−_ constitutes the random part of the model. More precisely, *X*_*ij*_ is a vector of individual-level characteristics, *β* is a vector of *k* estimated parameter coefficients and *u*_*j*_ is the error term at the school-level which represents each school’s difference from the overall population mean as its mean is set to 0. *τ* represents the *precision* (1/variance) of the normal distribution of the response Hb whereas *τ*_*uj*_ represents the *precision* (1/variance) of the normal distribution of the *u*_*j*_’s. For both variance components, we follow the usual practice of specifying a gamma prior distribution to the corresponding precision parameters which is proper and close to being uniform on log (*τ*).

For the vector of the *k* estimated parameter coefficients we assumed:

$$p(\beta_{k})\propto \;1$$

Where *p*(*β*_*k*_) symbolizes the prior distribution of the *k* estimated parameters and is proportional to 1.

In order to examine the prevalence of anaemia with associated covariate vectors, we chose the single outcome of the probability that a child is anaemic as estimated by a hierarchical Bayesian logistic regression model. We labeled the survey responses *anaemia*_*ij*_ as 1 for children i being anaemic (if Hb < 11.0 g/dL) in school j and 0 otherwise, and model them independently with Pr(anaemia_*ij*_ = 1) = logit^−1^((X*β*)_*ij*_). We present an analysis based on a prior distribution for *β* that is independent and locally uniform in the *k* parameters; that is *p(β*_1_, …, *β*_*k*_) ∝ 1. Specifically for the risk of being anaemic, we used a random intercepts logit link model which can be written as:

$$\begin{array}{cc} & {\text{anaemia}}_{\mathrm{ij}}∼\text{binomial}(\pi_{\mathrm{ij}}\text{,}n_{\mathrm{ij}})\text{,}\\ & \mathrm{with}\;\mathrm{logit}(\pi_{\mathrm{ij}})=X_{\mathrm{ij}}\beta +u_{j}\;\mathrm{and}\;\tau ∼\text{gamma}(0.001\text{,}0.001)\text{,}\\ & u_{j}∼\mathrm{normal}(0\text{,}\tau_{u_{j}})\;\mathrm{and}\;\tau_{u_{j}}∼\text{gamma}(0.001\text{,}0.001) \end{array}$$

Where *n*_*ij*_ is the total number of events (this is equal to 1523 children). The rest of the notation remains identical to the normal hierarchical Bayesian models which were employed for the analysis of the Hb levels. In these aforementioned regression models we used the same predictors of anaemia prevalence as those in the models of Hb counts.

To compare model complexities and goodness of fit we also monitored the recently proposed deviance information criterion (DIC) of (Spiegelhalter et al., 2002).

## 3 Results

Data on Hb, malaria parasitaemia and helminth infection were available for 1523 children (aged 10–21 years) in the 30 schools (Table 1). Overall 13.5%, (95% Confidence Interval (CI): 11.8–15.2) of these children were anaemic at the time of survey and the mean Hb concentration was estimated to be 12.43 g/dL, (95% CI: 12.35–12.50).

A total of 34.7% of children were infected with *Plasmodium falciparum* with a further 0.3% having mixed infections with *P. falciparum* and *Plasmodium malariae*. Up to 76.9% of children were infected with at least one parasitic helminth infection. Hookworm was the most prevalent helminth infection (47.3%); 14.1% were infected with *S. mansoni*, 23.7% with *A. lumbricoides* and 12.9% with *T. trichiura.* The prevalences of *P. falciparum*, hookworm and *S. mansoni* were, respectively, 35.8%, 41.2% and 12.4% in the 10–12 years old age group, 34.4%, 51.2% and 14.7% in the 13–15 years old age group and 25.9%, 46.3% and 20.4% in the older age group (i.e. > = 16 years old).

### 3.1 Bayesian hierarchical normal model of haemoglobin

**Fig. 1:**
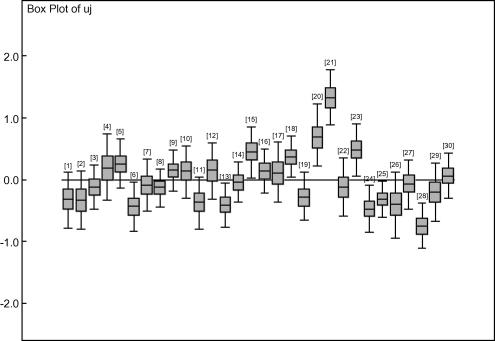
Box plot of school-level residuals from a Bayesian hierarchical model for Hb counts among older children. Each box plot represents a school-level residual uj in our study. Numbers above each box plot are label identifiers for each school. This is a plot in which the posterior distributions of all ujs are summarised side by side. Boxes represent inter-quartile ranges and the solid black line at the (approximate) centre of each box is the mean of each specific uj; the arms of each box extend to cover the central 95 percent of the distribution–their ends correspond, therefore, to the 2.5% and 97.5% quantiles. The horizontal straight line in the middle of the graph represents the overall mean of the ujs which is set to 0.

Table 2 contains the posterior means and 95% credible intervals of the final two-level Bayesian normal hierarchical model for the Hb of children with complete data on all covariates. According to this model, the posterior mean for the overall mean Hb was estimated to be 12.52 g/dL (95% credible interval: 12.28–12.76). On average, girls had lower mean Hb compared with boys by 0.18 g/dL. Older children tended to have higher mean Hb than children aged 10–12 years. Stunted children compared with non-stunted children had lower mean Hb by 0.34 g/dL. From the 95% credible intervals of all the parasitic infections, only children heavily infected with *S. mansoni* had significantly lower mean Hb by an average of 0.51 g/dL, (95% credible interval: −0.94 to −0.10) and children infected with malaria had significantly lower mean Hb by an average of 0.16 g/dL, (95% credible interval: −0.32 to 0.01) compared with uninfected children, respectively. Although there was no evidence of a significant effect of the intensities of single hookworm, *T. trichiura* or *A. lumbricoides* infection, we still allowed adjustment for these and therefore they were finally included in the model. The two-way interaction terms of intensities of helminth infections mentioned before, were also included and tested in the models in order to check for the effect of helminth co-infections on the mean Hb. As none of these terms, as well as the SES, were found to be significantly associated with Hb levels and/or anaemia, they were omitted from the final model. The random effects variance components indicate that most of the variance is between children within a school: of the total variability in Hb, 9.9% (0.225/2.256) occurred at the school level while 90.0% (2.031/2.256) occurred between children within a school. This is also illustrated by Fig. 1 which presents a box plot of the level-2 residuals among older children for each school, and indicates that most schools had similar Hb levels that could not be distinguished statistically, thereby confirming the suitability of assumptions for the chosen final model. Interestingly, mean Hb was substantially higher in one school (#21) relative to other schools.

### 3.2 Bayesian logistic regression model of anaemia

Table 3 presents the hierarchical logistic regression model of anaemia risk and shows that only children with heavy *S. mansoni* intensities were more likely to be anaemic, defined as Hb < 11.0 g/dL, compared with uninfected children (Odds ratio: OR = 2.3, 95% credible interval: 1.1–4.3; Table 3). There was no evidence that other predictors were significantly associated with the risk of anaemia.

## 4 Discussion

Malaria, undernutrition and helminth infections have a large impact on the survival and quality of lives of school-aged children living in Africa. Understanding the direct and indirect consequences of these factors on lower Hb levels and anaemia is important, as findings may help guide the suite of school-based interventions in endemic areas where polyparasitism is the norm (Raso et al., 2004; Pullan and Brooker, 2008). Our analysis found evidence that malaria parasitaemia, heavy intensity of *S. mansoni* infection and being stunted were significantly associated with lower mean Hb, although only heavy intensity of *S. mansoni* infection was significantly associated with the risk of anaemia among schoolchildren over 10 years of age. Such results underscore current efforts to control helminths and malaria as part of integrated school health programmes (Bundy et al., 2006; Brooker et al., 2008).

Although our cross-sectional design hampers the interpretation of our finding, especially the direction of causality, the use of hierarchical Bayesian modelling allows the incorporation of both individual- and school-level factors, the omission of one or the other leading to biased estimates (Congdon, 2001). Furthermore, our results are consistent with previous studies which report similar associations (Stoltzfus et al., 1997b; Olsen et al., 1998; Leenstra et al., 2004; Desai et al., 2005a). However, the difference in Hb between children infected with malaria and those uninfected, though significant, was small. The anaemia of malaria is multifactorial, involving a complexity of mechanims including increased destruction of red blood cells (RBCs) through rupturing, phagocytosis and hypersplenism, and decreased RBC production through inflammation and dyserythropiesis (Menendez et al., 2000). The single time point of our cross-sectional design means that we were unable to capture information on clinical attacks of malaria and therefore separate haemolysis due to clinical malaria and the role of asymptomatic low grade infections on RBC concentrations and Hb. Neither can this design examine the effect of recently cleared infections on the process of haematological recovery and current Hb. However, clearer evidence of the contribution of malaria to anaemia in school-aged children is provided by the results of our intervention trial which showed that school-based intermittent preventive treatment with sulfadoxine-pyrimethamine and amodiaquine significantly reduced the prevalence of anaemia among the wider age range of schoolchildren included in the main trial (Clarke et al., 2008).

A surprising finding was the strong association between heavy intensity of *S. mansoni* infection, anaemia and Hb. Potential mechanisms through which *S. mansoni* may contribute to anaemia include: (i) blood loss caused by the rupture of blood vessels surrounding the intestine by the spined schistosome eggs; (ii) splenic sequestration; (iii) autoimmune hemolysis; and (iv) anaemia of inflammation which is typically characterised by decreased RBC production induced by pro-inflammatory cytokines (Friedman et al., 2005a; Tolentino and Friedman, 2007). In addition it is possible that the importance of *S. mansoni* infection is likely to be greater than estimated here since only schools located more than 5 km from the lake shore were sampled to purposively minimise confounding by *S. mansoni* in the intervention trial. By contrast, hookworm infection, a major attributable factor for anemia in schoolchildren in other areas of East Africa (Stoltzfus et al., 1997a; Lwambo et al., 2000), was not associated either with lower Hb or with anemia in the present study. This finding, which is consistent with previous studies in western Kenya (Olsen et al., 1998; Handzel et al., 2003), is probably due in part to the low intensity of hookworm infection in our study area, and highlights how different factors contribute to anaemia in different parasite transmission settings. We did not find any evidence of an increased risk of anaemia in children co-infected with multiple helminth species and/or malaria.

The results of our Bayesian linear hierarchical approach indicate that a high degree of variation remains to be explained and that there are other factors beyond what was measured in this study which still need to be considered. One advantage of a hierarchical approach over conventional statistical approaches is the partitioning of the unexplained variance into variability between clusters and individual level variation within clusters, which shows that most of the residual variation within our study population was attributable to individual level variation occurring between children. More precisely, although dietary iron insufficiency is very likely to impact on Hb and anaemia (Olivares et al., 1999), no information was available on iron status of the children included in the study. Nutritional variables which were measured such as stunting and wasting, representing chronic and severe acute undernutrition, respectively, may not adequately capture moderate, current undernutrition which may also explain variation in mean Hb levels. Reported age is also often uncertain and may be inaccurate. This may have implications for the reliability of the derived HAZ and BMIZ scores. A further source of variation not measured in this study is the effect of menarche in adolescent girls (Leenstra et al., 2004). When interaction terms of age and sex were fitted to the model these were not found to be significant, however as described above, reported age may be both unreliable and too crude a proxy for individual variation in the timing of onset of menarche. Similarly, the SES index used here may not fully capture socio-economic variability within the population as it was based on a small number of assets and relied on reporting by schoolchildren. A final source of individual variation which was not taken into account in this study was genetic traits such as sickle-cell and other haemoglobinopathies (Tolentino and Friedman, 2007).

In conclusion, this study demonstrated that lower mean Hb levels were significantly associated with malaria, chronic undernutrition and heavy intensity of *S. mansoni*, and that anaemia was associated with heavy intensity of *S. mansoni*. Such results have important implications for the control of anaemia among African schoolchildren and can help guide the design of appropriate interventions. Integrated school health programmes which include deworming, micronutrients and potentially malaria control, will help alleviate the anaemia burden faced by the school-aged children of Africa. Further research is required to identify the optimal packages and to identify areas where different packages of interventions may be required.

## Figures and Tables

**Table 1:** Mean Hb concentration and prevalence of anaemia according to infection status and other characteristics in 1523 schoolchildren aged 10–21 years (univariate analysis)

Variable	Children No (%)	Mean Hb level, g/dL (95% CI)	Anaemia prevalence (%) (95% CI)
Sex			
Male	786 (51.6)	12.51 (12.40–12.61)	13.2 (10.9–15.6)
Female	737 (48.4)	12.34 (12.23–12.45)	13.7 (11.2–16.2)
Age			
10–12 years old	573 (39.0)	12.29 (12.17–12.41)	13.1 (10.3–15.9)
13–15 years old	896 (61.099)	12.52 (12.42–12.62)	13.8 (11.6–16.1)
> = 16 years old	54 (3.7)	12.44 (12.08–12.80)	11.1 (2.7–19.5)
Classification of SES (*n* = 1404^b^)			
Least poor	281 (20.01)	12.42 (12.24–12.61)	12.81 (8.90–16.72)
Poor	560 (39.89)	12.45 (12.34–12.58)	13.93 (11.06–16.79)
Most poor	563 (40.10)	12.40 (12.28–12.53)	13.14 (10.35–15.93)
Classification of BMIZ			
Not underweight	1430 (93.9)	12.44 (12.36–12.52)	13.2 (11.4–14.9)
Underweight	93 (6.1)	12.22 (11.92–12.52)	18.3 (10.4–26.1)
Classification of HAZ			
Not stunted	1323 (86.9)	12.48 (12.40–12.56)	12.8 (11.0–14.6)
Stunted	200 (13.1)	12.10 (11.88–12.31)	18.0 (12.7–23.3)
Intensity of hookworm infection^c^			
Not infected	803 (52.7)	12.42 (12.31–12.53)	14.0 (11.6–16.3)
Lightly infected	691 (45.4)	12.45 (12.35–12.56)	12.9 (10.4–15.4)
Moderately infected	15 (1.0)	12.21 (11.60–12.83)	13.3 (0.0–30.5)
Heavily infected	14 (0.9)	11.87 (11.34–12.40)	14.3 (0.0–32.6)
Intensity of *S. mansoni* infection^*c*^			
Not infected	1309 (86.0)	12.44 (12.36–12.53)	13.2 (11.4–15.1)
Lightly infected	91 (6.0)	12.41 (12.11–12.71)	11.0 (4.6–17.4)
Moderately infected	76 (5.0)	12.54 (12.21–12.87)	13.2 (5.6–20.8)
Heavily infected	47 (3.0)	11.79 (11.28 to 12.30)	25.5 (13.1–38.0)
Intensity of *T.trichiura* infection^c^			
Not infected	1326 (87.1)	12.41 (12.33–12.50)	13.4 (11.6–15.3)
Lightly infected	188 (12.3)	12.47 (12.26–12.69)	14.4 (9.4–19.4)
Moderately infected	8 (0.5)	13.26 (12.30–14.23)	0.0 NA^a^
Heavily infected	1 (0.1)	12.80 NA^a^	0.0 NA^a^
Intensity of *A. lumbricoides* infection^c^			
Not infected	1162 (76.3)	12.46 (12.37–12.55)	13.6 (11.6–15.6)
Lightly infected	236 (15.5)	12.34 (12.16–12.52)	13.1 (8.8–17.5)
Moderately infected	125 (8.2)	12.27 (12.04–12.50)	12.8 (6.9–18.7)
Heavily infected	0 (0.0)	0.0 NA^a^	0.0 NA^a^
Malaria spp. infection			
Not infected	989 (69.9)	12.49 (12.40–12.58)	12.8 (10.8–14.9)
Infected	534 (35.1)	12.31 (12.18–12.44)	14.6 (11.6–17.6)

BMIZ, Body Mass Index *Z*-score; HAZ, Height for Age *Z*-score; CI, confidence interval.

a NA, not available.

b Socio-economic status (SES) data were missing for 119 children.

c Intensity of helminth infection was classified in light, moderate and heavy according to WHO recommended thresholds: *Schistosoma mansoni* infection, 1–99, 100–399 and ⩾400 eggs per gram of faeces (epg); hookworm, 1–1999, 2000–3999 and ⩾4000 epg; *Trichuris trichiura*, 1–999, 1000–9999 and ⩾10,000 epg; and *Ascaris lumbricoides*, 1–4999, 5000–49,999 and ⩾50,000 epg.

**Table 2:** Estimated posterior mean differences in mean Hb concentration for the effects of selected explanatory variables from a final Bayesian hierarchical model (*n* = 1523)

Variable	Mean	95% credible interval^a^
Fixed part of the model		
Intercept	12.520	(12.280–12.760)
Sex (Reference category: ‘Male’) Female	−0.183	(−0.330 to −0.036)^b^
Age (Reference category: ‘10–12 years old’)		
13–15 years old	0.222	(0.064–0.377)^b^
> = 16 years old	0.417	(0.012–0.832)^b^
Classification of BMIZ (Reference category:’ Not wasted’) Wasted	−0.244	(−0.544 to 0.062)
Classification of HAZ (Reference category:’ Not stunted’) Stunted	−0.347	(−0.564 to −0.128)^b^
Intensity of hookworm infection (Reference category: ‘Not Infected’)		
Lightly infected	0.050	(−0.102 to 0.197)
Moderately infected	−0.310	(−1.048 to 0.419)
Heavily infected	−0.516	(−1.277 to 0.233)
Intensity of *Schistosoma mansoni* infection (Reference category: ‘Not Infected’)		
Lightly infected	−0.113	(−0.417 to 0.189)
Moderately infected	0.068	(−0.268 to 0.414)
Heavily infected	−0.513	(−0.942 to −0.097)^b^
Intensity of *Trichuris trichiura* infection (Reference category: ‘Not Infected’)		
Lightly infected	0.105	(−0.121 to 0.336)
Moderately infected	0.834	(−0.195 to 1.857)
Heavily infected	0.110	(−2.649 to 2.890)
Intensity of *Ascaris lumbricoides* infection (Reference category: ‘Not Infected’)		
Lightly infected	−0.164	(−0.372 to 0.047)
Moderately infected	−0.206	(−0.480 to 0.073)
Malaria spp infection (Reference category: ‘Not Infected’) Infected	−0.159	(−0.315 to −0.009)^b^
Random part of the model		
Level-2 (i.e. between schools) variance	0.225	(0.114–0.413)
Level-1 (i.e. between children within a school) variance	2.031	(1.889–2.186)

BMIZ, Body Mass Index *Z*-score; HAZ, Height for Age *Z*-score.

a 95% Credible intervals (CIs) are different from classical 95% confidence intervals in various ways, some of which are: (i) in their interpretation: we say there is a 95% probability that the true parameter lies in a 95% credible interval where this is certainly not the interpretation of a 95% confidence interval. In a long series of 95% confidence intervals, 95% of those should contain the true parameter value – unlike the Bayesian interpretation we cannot give a probability for whether a particular confidence interval contains the true value; and (ii) credible intervals will generally be narrower due to the additional information provided by the prior (Spiegelhalter et al., 2004).

b These are significant differences compared with the reference category in the sense that the probability is at least 95% that these parameters lie within the credible interval, which is significant.

**Table 3:** Estimated posterior odds ratios for prevalence of anaemia (Hb < 110 g/L) for the effects of selected explanatory variables from final Bayesian hierarchical logistic regression model (*n* = 1523)

Variable	Odds ratio	95% credible interval
Fixed part of the model		
Main effects		
Sex (Reference category: ‘Male’)		
Female	1.073	(0.787 to 1.405)
Age (Reference category: ‘ > =10–12 years old’)		
13–15 years old	1.095	(0.804–1.462)
> = 16 years old	0.688	(0.218–1.547)
Intensity of hookworm infection (Reference category: ‘Not Infected’)		
Lightly infected	0.895	(0.645–1.208)
Moderately infected	1.156	(0.121–3.708)
Heavily infected	1.165	(0.132–3.861)
Intensity of *Schistosoma mansoni* infection (Reference category: ‘Not Infected’)		
Lightly infected	0.825	(0.360–1.442)
Moderately infected	1.004	(0.426–1.969)
Heavily infected	2.292	(1.070–4.258)^a^
Intensity of *Ascaris lumbricoides* infection (Reference category: ‘Not Infected’)		
Lightly infected	1.060	(0.680–1.584)
Moderately infected	1.057	(0.582–1.748)
Malaria spp infection (Reference category: ‘Not Infected’) infected	1.136	(0.821–1.540)
*Random part of the model*		
Level-2 (i.e. between schools) variance	0.288	(0.088–0.630)

a This is a significant odds ratio of heavily infected children with *S. mansoni* compared with uninfected children in the same sense as denoted in Table 2.
